# Management of Juvenile Fibromyalgia: A Level I Evidence-Based Systematic Review

**DOI:** 10.3390/medsci13030203

**Published:** 2025-09-22

**Authors:** Filippo Migliorini, Nicola Maffulli, Michael Kurt Memminger, Francesco Simeone, Tommaso Bardazzi, Maria Grazia Vaccaro, Giorgia Colarossi

**Affiliations:** 1 Department of Trauma and Reconstructive Surgery, University Hospital of Halle, Martin-Luther University Halle-Wittenberg, 06097 Halle (Saale), Germany; 2 Department of Orthopaedic and Trauma Surgery, Academic Hospital of Bolzano (SABES-ASDAA), Via Lorenz Böhler 5, 39100 Bolzano, Italy; michael.memminger@sabes.it (M.K.M.); francesco.simeone@sabes.it (F.S.); tommaso.bardazzi@sabes.it (T.B.); 3 Department of Life Sciences, Health, and Health Professions, Link Campus University, Via del Casale di San Pio V, 00165 Rome, Italy; 4 Faculty of Medicine and Psychology, University “La Sapienza” of Rome, 00185 Rome, Italy; n.maffulli@qmul.ac.uk; 5 School of Pharmacy and Bioengineering, Faculty of Medicine, Keele University, Stoke on Trent ST4 7QB, UK; 6 Centre for Sports and Exercise Medicine, Barts and the London School of Medicine and Dentistry, Mile End Hospital, Queen Mary University of London, London E1 4DG, UK; 7 Neuroscience Research Centre, Department of Medical and Surgical Sciences, Magna Graecia University, 88100 Catanzaro, Italy; mg.vaccaro@unicz.it; 8 Department of Cardiology, Rhein-Maas Teaching Hospital, 52146 Wuerselen, Germany; giorgia.colarossi@gmail.com

**Keywords:** multidisciplinary care, chronic pain, mood disorders, functional impairment, adolescent health, treatment outcomes, cognitive behavioural therapy

## Abstract

Background: Juvenile fibromyalgia (JFM) is a chronic pain disorder characterised by widespread musculoskeletal pain, functional impairment, fatigue, and mood disturbances. Treatment remains challenging, considering the multifactorial nature of the condition and the limited high-quality evidence supporting pharmacological or non-pharmacological interventions. Objectives: This review aimed to critically appraise level I evidence from randomised controlled trials assessing the efficacy and safety of pharmacological and non-pharmacological treatments for adolescents with JFM. Methods: Seven published peer-reviewed clinical trials were examined, including studies investigating duloxetine, milnacipran, pregabalin, cognitive-behavioural therapy (CBT), and the integrated Fibromyalgia Integrative Training Teens (FIT) program, which combines CBT with neuromuscular training. Outcomes of interest included pain intensity, functional disability, depression symptoms, physical activity, and adverse events. Results: Pharmacological agents such as duloxetine, milnacipran, and pregabalin demonstrated modest improvements in pain, but failed to produce consistent benefits in function or mood, and were associated with a high incidence of adverse effects. CBT significantly improved functional disability and depression symptoms, yet it had a limited impact on pain reduction or objectively measured activity levels. The FIT Teens program showed superior outcomes in pain intensity and biomechanical function compared to CBT alone, suggesting a synergistic effect of combining psychological and physical reconditioning strategies. Conclusions: Current evidence supports the use of multimodal treatment approaches in JFM. Non-pharmacological interventions, particularly when integrated with structured exercise, offer meaningful benefits with minimal safety concerns. Larger, methodologically rigorous trials are needed to establish optimal treatment pathways and long-term outcomes for this complex and underserved paediatric population.

## 1. Introduction

Juvenile fibromyalgia (JFM) is a chronic pain syndrome that affects up to 6% of school-aged children and adolescents, with the most common age of onset between 11 and 15 years [1,2]. JFM is most frequently diagnosed around the time of puberty, with a higher prevalence in females aged 11.4 to 13.7 years [3]. Although the clinical manifestations are similar to those in adults, the management of JFM is controversial, and evidence is lacking [4,5,6,7]. Widespread pain, fatigue, sleep disturbance, and physical and psychological impairment are common, leading to impaired daily activities, school achievements, and social functioning [8,9,10]. In addition, joint laxity or hypermobility and specific psychological manifestations, including depression and anxiety, are peculiar in JFM [4]. The aetiology of JFM is not fully understood, but a genetic predisposition is likely [11,12,13]. Physical or psychological trauma and illnesses may precede the occurrence of the symptoms, acting as possible triggers [11,14]. Similar to the adult form, abnormality in pain processing and perception is involved [3,15]. The management of JFM is challenging, and establishing an effective and well-tolerated treatment plan is essential. However, evidence of the efficacy and safety of pharmacological management remains limited [7,16,17,18,19]. Pregabalin is primarily an anticonvulsant, which inhibits calcium channels, thereby reducing presynaptic neurotransmitter release and postsynaptic excitability [20,21]. Given its effects on central pain modulation, it was also approved for the treatment of adults with fibromyalgia [22]. Duloxetine and milnacipran are serotonin-norepinephrine reuptake inhibitors (SNRIs), which work by increasing serotonin and norepinephrine levels and enhancing the descending inhibitory pain pathways [23,24,25,26]. Adolescents have a limited variety of available treatments, and drugs approved by the Food and Drug Administration (FDA) for the management of fibromyalgia in adults are not approved for JFM [27]. Pregabalin, duloxetine and milnacipran are currently approved for the treatment of fibromyalgia in adults. A multidisciplinary approach, including cognitive behavioural therapy and physical exercises, is recommended for JFM. Cognitive behavioural therapy (CBT) aims to train patients in the use of specific cognitive and behavioural techniques to enhance pain coping skills and decrease pain-related functional impairment [28]. However, the efficacy of non-pharmacological approaches in JFM is still controversial. Recently, randomised controlled trials (RCTs) evaluating pharmacological and non-pharmacological approaches in JFM have been published [29,30,31,32,33,34,35,36]. A comprehensive systematic review assessing the efficacy of a non-pharmacological approach in combination with standard pharmacological management in JFM is still lacking.

This systematic review evaluated the efficacy of pharmacological and non-pharmacological management in JFM. The outcomes of interest were patient-reported outcome measures (PROMs), the rate of adverse events, and the rate of adolescents who discontinued the treatment regimen.

## 2. Materials and Methods

### 2.1. Eligibility Criteria

All the RCTs comparing the efficacy of pharmacological and non-pharmacological management of JFM were accessed. According to the author’s language capabilities, articles written in English, German, Italian, French, and Spanish were eligible for consideration. According to the Oxford Centre of Evidence-Based Medicine [37], only studies with level I evidence were considered. Reviews, opinions, letters, and editorials were not considered. Only studies which evaluated drugs administered orally were considered. Studies which investigated the administration of opioids, non-steroidal anti-inflammatory drugs (NSAIDs), or injection therapy were not considered. Only studies conducting a minimum of eight weeks of follow-up were eligible. Only studies that included adolescents younger than 18 years were considered for inclusion. Only studies which reported quantitative data were considered. Only data from parallel studies were considered. The missing quantitative data on the outcomes of interest warranted the exclusion of the study.

### 2.2. Search Strategy

This study was conducted according to the Preferred Reporting Items for Systematic Reviews and Meta-Analyses: the 2020 PRISMA statement [38]. The PICOTD framework was established:

- P (Population): JFM;
- I (Intervention): Pharmacological and non-pharmacological management;
- C (Comparator): Not applicable;
- O (Outcome): PROMs, adverse events and therapy discontinuation.
- T (Timing): minimum 8 weeks of follow-up
- D (Design): RCT.

In January 2025, PubMed, Web of Science, and Embase were accessed with no time constraint. The keywords used for the search using the Boolean operators AND/OR are reported in Table 1. The search was restricted to RCTs only.

### 2.3. Selection and Data Collection

Two authors (T.B. and F.S.) independently performed the database search. All the resulting titles were screened by hand, and the abstract was accessed if suitable. The full text of the abstracts that matched the topic of interest was accessed. If the full text was not accessible or available, the article was excluded from consideration. A cross-reference of the bibliography of the full-text articles was also performed for inclusion. Disagreements were debated and mutually solved by the authors. A third senior author (N.M.) made the final decision in case of further disagreement.

### 2.4. Data Items

Two authors (T.B. and F.S.) independently performed data extraction. The baseline data were extracted, including author, year of publication, journal, length of follow-up, number of women, number of patients, and their related mean age. Data concerning the following PROMs were collected at baseline and at last follow-up: Children’s Depression Inventory (CDI) [39], Functional Disability Inventory (FDI) [40], and Visual Analogue Scale (VAS). The rate of adolescents who discontinued therapy and the adverse events were also investigated.

### 2.5. Assessment of the Risk of Bias and Quality of the Recommendations

The risk of bias was assessed according to the guidelines in the Cochrane Handbook for Systematic Reviews of Interventions [41]. Two reviewers (J.E. and M.K.M.) independently evaluated the risk of bias in the extracted studies. Disagreements were solved by a third senior author (N.M.). RCTs were evaluated using the risk of bias tool in Review Manager 5.3 (The Nordic Cochrane Collaboration, Copenhagen, Denmark). The following endpoints were evaluated: selection, detection, performance, attrition, reporting, and other biases.

### 2.6. Synthesis Methods

The main author (F.M.) performed the statistical analyses following the recommendations of the Cochrane Handbook for Systematic Reviews of Interventions [42]. Mean and standard deviation were used for descriptive statistics.

## 3. Results

### 3.1. Study Selection

**Figure 1:**
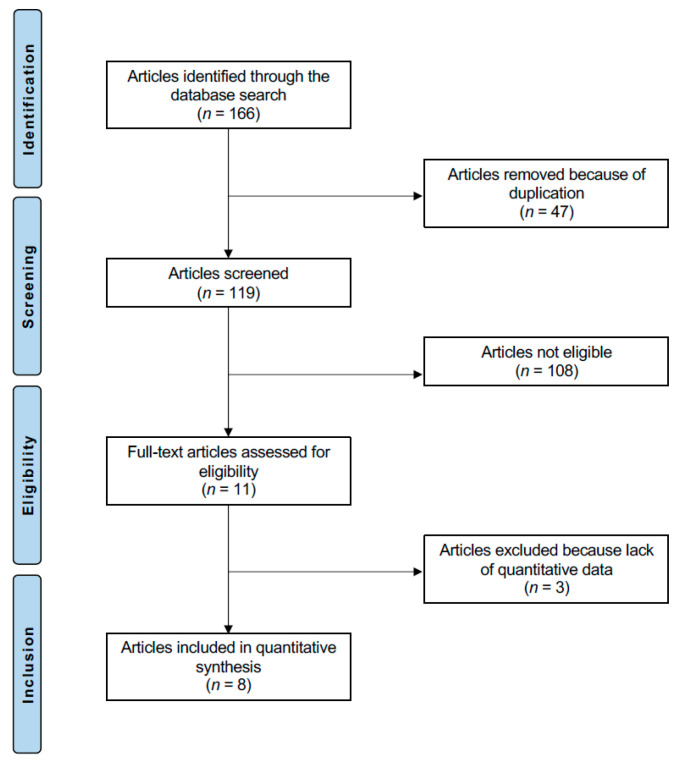
PRISMA flow chart of the literature search.

The literature search identified 166 articles. Of them, 47 were duplicates and, therefore, excluded. An additional 108 studies were excluded: study design (*N* = 98), duration of the follow-up shorter than 8 weeks (*N* = 5), matching JFM with healthy control (*N* = 1), single-arm studies (*N* = 3), and investigation of the adult form of fibromyalgia (*N* = 1). Eleven articles were assessed for eligibility. An additional three studies were excluded as they did not report data on the outcomes of interest. This left eight studies for the present study. The results of the literature search are shown in Figure 1.

### 3.2. Risk of Bias Assessment

**Figure 2:**
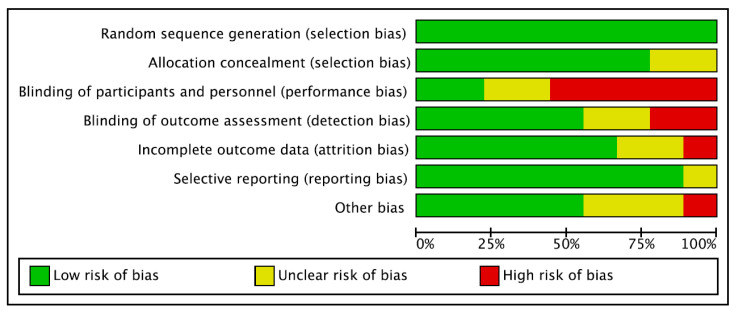
Cochrane risk of bias tool.

Given the appropriate randomisation sequence generation and the balanced allocation concealment, the risk of selection bias was low. In four of the included studies, a double-blinded phase was followed by an open-label phase. Patients were not blinded in most of the included studies, and blinding of assessors was often performed, leading to a moderate to high risk of performance bias and a low to moderate detection bias. The overall risk of attrition and reporting bias was low to moderate, as was the risk of other biases. Concluding, the risk of bias graph evidenced a good quality of the methodological assessment (Figure 2).

### 3.3. Study Characteristics and Results of Individual Studies

Data from 849 patients were included for analysis. The mean length of follow-up was 12.8 ± 5.2 weeks. The mean age was 15.2 ± 0.4 years. Table 2 presents the general characteristics of the included studies, while Table 3 provides an overview of the studies included.

### 3.4. Results Syntheses

In adolescents with juvenile fibromyalgia, pharmacological management has not shown consistent primary analgesic benefits, whereas psychological and neuromuscular approaches have yielded clearer functional gains (Table 3). Arnold et al. [29], in an open-label study evaluating milnacipran 50–100 mg daily, reported mean improvements in pain, global severity, quality of life, and fatigue, along with nausea, headache, vomiting, and dizziness, as well as small increases in heart rate and blood pressure. In another placebo-controlled trial by the same study group [30], the administration of pregabalin at 75–450 mg daily did not improve the primary endpoint, the mean pain score at endpoint. However, weekly pain trajectories and patient global impression of change favoured pregabalin, with a safety profile consistent with that of adults. Upadhyaya et al. [36] found no advantage of duloxetine 30–60 mg daily over placebo for 24-h average pain, while responder rates and selected interference items favoured duloxetine, and adverse events were more frequent. Hengartner et al. [32] reported a higher incidence of severe psychiatric adverse events, including suicidality, with duloxetine. On the non-pharmacological side, Kashikar-Zuck et al. [33] demonstrated that cognitive behavioural therapy was superior to education in reducing functional disability without significant changes in pain. A subsequent investigation by the same group [34] observed improved self-reported function without parallel gains on actigraphy. More recently, Kashikar-Zuck [35] and Black [31] found that the FIT Teens programme produced larger pain reductions than cognitive behavioural therapy and improved hip strength and movement patterns, with disability gains not consistently maintained at three months.

## 4. Discussion

### 4.1. Pathophysiology and Treatment Challenges in Juvenile Fibromyalgia

JFM is a chronic and debilitating condition characterised by diffuse musculoskeletal pain, fatigue, sleep disturbances, and mood symptoms, primarily affecting adolescent females [11,43,44,45,46,47,48,49,50,51]. The pathophysiological mechanisms underpinning JFM are not yet fully elucidated; however, central sensitisation plays a pivotal role, resulting in augmented nociceptive processing and hypersensitivity to both painful and non-painful stimuli [19,52,53,54,55,56,57,58,59,60]. Neurobiological dysfunctions in descending pain inhibitory pathways, along with psychosocial contributors such as anxiety, catastrophising, and family dynamics, further complicate disease expression and perpetuation [61,62,63,64,65,66,67]. Despite the considerable burden of disease, there are currently no medications formally approved for the treatment of JFM. Clinical management is therefore guided by extrapolated data from adult fibromyalgia trials and a limited body of paediatric research [68,69,70,71,72,73,74,75,76,77,78,79,80,81,82,83,84,85]. The condition remains underdiagnosed, and diagnostic latency is frequently encountered, with patients often undergoing extensive medical evaluation before a formal diagnosis is established [3,11,16,49,50,51,86,87,88]. Moreover, the multi-symptomatic nature of JFM poses significant therapeutic challenges, as effective management requires not only a reduction in pain intensity but also improvement in function, sleep, mood, and physical activity engagement. Pharmacological treatments, including duloxetine, milnacipran, and pregabalin, have been studied in adolescents with JFM with mixed results. While these agents target central pain mechanisms and have demonstrated efficacy in adult fibromyalgia populations, their application in younger cohorts has yielded only partial or inconclusive outcomes [89,90,91,92,93,94,95,96,97,98,99,100]. Furthermore, concerns regarding tolerability and psychiatric side effects, particularly in the case of serotonin-norepinephrine reuptake inhibitors (SNRIs), have limited their widespread adoption in paediatric practice. Non-pharmacological therapies, particularly CBT, have shown promising results in improving functional disability and mood, yet they often fail to produce clinically significant reductions in pain [101,102,103,104,105]. The lack of a single, clearly superior intervention highlights the complexity of JFM and underscores the need for an integrative, multimodal treatment paradigm. This has prompted growing interest in combined therapeutic strategies that can address both the somatic and psychosocial dimensions of the condition, potentially leading to more sustainable clinical benefits.

### 4.2. Pharmacological Therapies: Efficacy and Limitations

The pharmacological management of JFM remains a matter of debate, with available evidence offering limited support for its standalone use in adolescents [27]. Three agents (duloxetine, milnacipran, and pregabalin) have received regulatory approval for the management of adult fibromyalgia, but are not formally licensed for paediatric populations [14,97,99,100,106,107,108,109,110,111,112,113]. Nevertheless, these medications have been evaluated in RCTs involving adolescents, albeit with methodological constraints and inconsistent outcomes. In a phase 3b RCT comparing duloxetine 30–60 mg daily with placebo over 13 weeks, no statistically significant difference was found in the primary outcome, namely the change in 24-h average pain severity on the Brief Pain Inventory (BPI). However, secondary analyses revealed that a significantly higher proportion of participants receiving duloxetine achieved clinically meaningful reductions in pain intensity (≥30% and ≥50%), and improvement in functional interference items, such as general activity and interpersonal relationships. Despite these findings, the duloxetine group exhibited a higher incidence of treatment-emergent adverse events (82.4% vs. 62.3%), including nausea, headache, and mood-related symptoms, raising concerns about tolerability and safety in adolescent patients [36]. Milnacipran, another serotonin-norepinephrine reuptake inhibitor (SNRI), was evaluated in a responder-enriched, randomised withdrawal trial. Given the insufficient recruitment, the study was unable to complete the double-blind phase, and formal efficacy analyses were not performed. Nonetheless, open-label observations from 116 participants indicated improvements in pain, global functioning, quality of life, and anxiety symptoms. Notably, the safety profile was consistent with that of adult populations, with the most common adverse events including nausea, dizziness, and transient cardiovascular effects [29]. These preliminary findings suggest potential benefits, but underscore the need for adequately powered RCTs with rigorous methodology and longer follow-up durations. Pregabalin, an α2δ ligand that modulates calcium channel activity [114], was assessed in a 15-week RCT with a 6-month open-label extension. While the primary endpoint, a reduction in mean pain score, was not met (*P* = 0.1), significant differences were observed at later timepoints (e.g., week 15) and in the global impression of change (PGIC), favouring pregabalin [30]. Notably, improvements in sleep and pain were more pronounced during the open-label phase. However, pregabalin failed to demonstrate efficacy in reducing functional disability or depressive symptoms. Its role in paediatric JFM thus appears limited to symptomatic pain relief, with minimal impact on broader quality-of-life domains. Taken together, current evidence suggests that pharmacological treatments may offer modest benefits in pain reduction, but often fall short in addressing functional impairment and psychosocial distress. Furthermore, the risk of adverse events, especially psychiatric effects in adolescents, must be carefully weighed. The lack of consistent efficacy across trials and the methodological variability limit the generalisability of findings. Consequently, pharmacological therapies should be considered within a broader, individualised, and multidisciplinary treatment framework, rather than as first-line monotherapy in this population.

### 4.3. Cognitive-Behavioural Therapy and Psychological Management

CBT has emerged as one of the most commonly studied non-pharmacological treatments for JFM, with multiple trials supporting its efficacy in improving functional outcomes and emotional well-being [101,102]. Unlike pharmacological agents, which primarily target central pain pathways, CBT addresses maladaptive cognitions and behaviours that contribute to pain-related disability and affective distress [103,104]. CBT aims to foster adaptive coping strategies, reduce catastrophising, and promote functional restoration by encouraging graded activity and behavioural activation [28]. In a large multisite RCT, CBT was significantly more effective than a structured fibromyalgia education programme in reducing functional disability, as measured by the FDI, both immediately after treatment and at 6-month follow-up [33]. Notably, while both CBT and education led to reductions in pain and depressive symptoms, the superiority of CBT was particularly evident in functional domains [33]. The observed improvements in depression symptoms were clinically meaningful, with most participants scoring within the non-depressive range by the end of the trial. However, pain reduction in both groups did not reach the threshold for clinical significance, with average decreases remaining below the 30% cut-off. These findings highlight an important distinction in treatment targets: while CBT appears to exert a robust effect on functional capacity and mood regulation, its direct impact on nociceptive pain appears more limited. This interpretation is further supported by actigraphy data collected in a subsequent study involving the same cohort, which revealed no significant increase in objectively measured physical activity following CBT, despite improvements in self-reported function [34]. This discrepancy between subjective functional gains and objective behavioural changes suggests that, while CBT enhances perceived competence and daily engagement, it may not substantially alter actual movement patterns or physical capacity without additional intervention. Given the multidimensional nature of JFM, the limitations of CBT in reducing pain intensity may reflect its focus on psychological adaptation rather than physiological modulation. Nevertheless, its safety profile, absence of pharmacological side effects, and durability of effects make CBT a cornerstone of JFM management. CBT has been especially effective in patients with prominent mood symptoms or high levels of pain-related fear and avoidance. Parental involvement, which is often integrated into CBT protocols, may further reinforce behavioural change and adherence. Ultimately, although CBT alone may be insufficient to address all aspects of JFM, particularly when pain is severe or refractory, it remains an essential component of a multimodal treatment strategy. Its integration with physical rehabilitation and carefully selected pharmacotherapy may offer the best chance for holistic improvement in this complex and vulnerable population.

### 4.4. Multimodal Approaches: The Role of FIT Teens

The persistent limitations of pharmacological and psychological monotherapies in JFM have prompted a growing interest in integrative models of care. One of the most promising innovations in this field is the FIT Teens programme, which combines cognitive-behavioural therapy with a structured neuromuscular exercise regimen [31,35,46,87,115]. This dual approach seeks to simultaneously address both the psychological and biomechanical contributors to pain and disability in adolescents with JFM. The FIT Teens protocol was developed as a group-based intervention, integrating standard CBT modules with progressive, developmentally tailored neuromuscular training [46]. Unlike conventional aerobic or resistance-based programmes, the exercise component is derived from injury-prevention paradigms used in sports medicine, focusing on core stability, movement biomechanics, and postural control [16]. Exercises are deliberately sequenced, advancing from isometric stabilisation to complex functional tasks to minimise delayed onset muscle soreness and promote adherence. In a pilot RCT comparing FIT Teens to CBT alone, participants in the integrated intervention exhibited significantly greater reductions in pain intensity at three-month follow-up, as well as improvements in functional disability scores [35]. Both groups demonstrated benefits, but the magnitude of change in pain was more pronounced in the FIT Teens arm, suggesting that the addition of neuromuscular training enhanced the overall efficacy of the psychological intervention. Interestingly, although improvements in disability did not statistically differ between groups at endpoint, a favourable trend was observed, and post hoc analyses revealed a consistent pattern of superiority for the combined protocol [35]. Beyond symptom scores, a secondary analysis from the same trial demonstrated biomechanical improvements in the FIT Teens group, including increased hip abduction strength and more favourable movement patterns during a drop vertical jump task, as assessed via 3D motion capture [31]. These adaptations are clinically relevant, as poor biomechanics have been associated with fear of movement, pain persistence, and functional impairment in chronic musculoskeletal conditions. The ability of FIT Teens to address these biomechanical deficits may explain its superior performance in reducing pain, especially when compared to CBT alone, which does not include targeted physical reconditioning. These findings support the hypothesis that effective JFM management requires not only cognitive restructuring but also physical re-education. By promoting correct movement strategies and enhancing proprioceptive control, neuromuscular training may reduce mechanical strain and movement-related fear, facilitating greater engagement in daily activities and potentially reversing the cycle of deconditioning. The integrated format also provides opportunities for in-session reinforcement of coping strategies during physical exertion, fostering skill generalisation and self-efficacy.

### 4.5. Functional Outcomes and Physical Activity Engagement

Functional impairment is a core component of JFM, with significant limitations reported in school attendance, physical activity, and participation in daily life [116]. Improvements in functional status are therefore a critical treatment target and a meaningful indicator of therapeutic success. While self-reported gains in function have been consistently observed following non-pharmacological interventions, objective measures of physical activity have yielded more nuanced insights. CBT has demonstrated robust effects in reducing functional disability, as captured by instruments such as the FDI. In the multisite RCT, CBT was significantly more effective than fibromyalgia education in reducing disability at post-treatment and at 6-month follow-up. However, subsequent analysis using actigraphy to monitor movement patterns revealed that these self-reported improvements were not paralleled by increases in actual physical activity [34]. Participants in both groups remained highly sedentary, and CBT recipients even showed significantly lower light and peak activity levels at post-treatment evaluation. These findings highlight a critical dissociation between perceived and objective function, suggesting that while CBT may enhance subjective confidence and behavioural coping, it does not necessarily translate into increased motor activity or fitness without an explicit physical component. The FIT Teens programme attempted to bridge this gap by combining CBT with neuromuscular exercise [117]. In addition to significant pain reduction and functional improvements, adolescents in the FIT Teens arm exhibited biomechanical adaptations suggestive of enhanced movement capacity, including improved hip strength and more stable postural mechanics during functional tasks. These changes are important, as poor strength and faulty biomechanics have been associated with fear of movement, avoidance behaviours, and reduced activity levels in adolescents with JFM [118]. By addressing these mechanical deficits, the integrated intervention may lower the perceived cost of movement, thus facilitating gradual re-engagement in physical activities. Moreover, the inclusion of motor re-education in FIT Teens aligns with emerging evidence suggesting that physical deconditioning, muscle imbalance, and altered neuromuscular control contribute to symptom persistence in chronic pain populations. Adolescents with JFM exhibit altered gait patterns, poor balance, and reduced lower limb strength, all of which may increase mechanical stress during movement and exacerbate pain responses. Interventions such as FIT Teens that target these deficits in a graded and developmentally appropriate manner may thus represent a key mechanism for restoring activity levels and functional independence. Despite these promising outcomes, no study to date has conclusively demonstrated that structured non-pharmacological interventions lead to objectively measured increases in free-living physical activity. Longitudinal assessments extending beyond 6 months and incorporating wearable activity monitors are needed to evaluate the durability of motor gains and behavioural generalisation. Moreover, future studies should investigate whether combining CBT with exercise may promote not only higher functional ability but also adherence to long-term activity regimens.

### 4.6. Safety Considerations and Adverse Events

In the management of JFM, safety and tolerability are of particular concern, especially given the chronicity of the condition, the young age of patients, and the complexity of pharmacological interventions. Adverse events (AEs), treatment-emergent side effects, and psychological risks must be carefully weighed when considering therapeutic options, particularly pharmacological agents that act on central pain processing mechanisms. Among the pharmacological treatments evaluated in adolescents with JFM, duloxetine has raised specific concerns. In a RCT, although duloxetine resulted in a higher proportion of patients achieving a clinically relevant reduction in pain (≥30% and ≥50%), a significantly higher rate of treatment-emergent AEs compared to placebo (82.4% vs. 62.4%, *p* = 0.003) was evidenced [36]. Reported adverse effects included nausea, headache, dizziness, and somnolence, as well as psychiatric symptoms. The risk of severe psychiatric adverse events, including suicidal ideation, remains a known concern with serotonin-norepinephrine reuptake inhibitors (SNRIs) in paediatric populations, and necessitates careful patient selection and monitoring. Milnacipran, another SNRI, exhibited a similar side effect profile in the open-label extension of its trial, with nausea, dizziness, vomiting, and increased blood pressure and heart rate [29]. Although no new safety signals were detected, the absence of a double-blind control group and the premature termination of the trial preclude firm conclusions about its risk-benefit profile in adolescents. Importantly, mean cardiovascular parameters increased during treatment, mirroring adult data and underscoring the need for cardiovascular surveillance in clinical practice. Pregabalin was evaluated in a 15-week placebo-controlled trial followed by a 6-month open-label extension [30]. While the overall safety profile was consistent with adult populations, with dizziness and somnolence being the most frequent AEs, the treatment did not significantly outperform placebo in primary pain outcomes. Consequently, despite relative tolerability, its modest efficacy limits its attractiveness as a monotherapy in paediatric settings. Furthermore, long-term use of anticonvulsants in adolescents raises theoretical concerns regarding neurocognitive development and requires further investigation. In contrast, non-pharmacological interventions, particularly CBT and integrative protocols such as FIT Teens, consistently demonstrated excellent safety profiles [31,33]. No serious adverse events were reported in studies of CBT alone or when CBT was combined with neuromuscular training. Minor musculoskeletal discomfort was occasionally noted during exercise, but resolved spontaneously and did not necessitate withdrawal from the intervention [35]. The high adherence rates and absence of dropouts related to safety concerns support the acceptability and tolerability of these interventions in adolescents. Furthermore, the FIT Teens programme incorporated deliberate pacing, tailored exercise intensity, and behavioural coaching to minimise the risk of symptom exacerbation. By gradually exposing participants to movement in a structured and supportive context, the intervention reduced fear of injury and promoted confidence in physical capability. This contrasts with the poor adherence frequently observed in unsupervised exercise regimens, which may provoke pain flares and reinforce avoidance.

### 4.7. Study Limitations and Methodological Heterogeneity

Several methodological shortcomings and a high degree of heterogeneity across studies limit the current evidence on the treatment of JFM. Although the Cochrane risk of bias tool indicated an overall acceptable methodological quality, moderate concerns regarding blinding and attrition should be acknowledged. These issues may have inflated placebo responses or led to incomplete outcome reporting, thereby reducing the certainty of the evidence and highlighting the need for more rigorous trial designs in this field. These limitations must be carefully considered when interpreting the findings and applying them to clinical practice. Of the seven studies included in the present review, three were conducted by the same research group, which may increase the risk of bias. The sample sizes in most RCTs remain modest, particularly in the pharmacological studies. For example, the milnacipran trial was terminated early from poor recruitment, failing to complete the double-blind withdrawal phase, and precluding statistical analysis of the primary endpoint. Similarly, the pregabalin trial enrolled 107 participants; while larger than previous efforts, the trial may have lacked sufficient power to detect small to moderate treatment effects on the primary outcome. The relatively small sample sizes increase the risk of type II error and limit the ability to conduct subgroup analyses or assess treatment moderators. There is significant variability in trial designs, including differences in inclusion criteria, diagnostic frameworks, outcome measures, and follow-up durations. Some studies used the Yunus and Masi criteria for JFM [30,32,33,34,36], while others adopted modified adult fibromyalgia criteria [29,31,35]. This lack of uniformity complicates cross-trial comparisons and may influence patient selection. Additionally, while most trials assessed pain and functional disability, the choice of primary outcomes varied, with some focusing on average pain scores and others on global impressions or functional indices. The time points for assessment also differed widely, ranging from 8 to 15 weeks, with limited long-term follow-up data beyond 6 months. A further concern lies in the under-reporting or inconsistent reporting of concomitant treatments. In several trials, patients undergoing non-pharmacological interventions were allowed to continue their existing medications, while others failed to clearly delineate whether pharmacological regimens were stable or modified during the study period. This lack of control over co-interventions introduces confounding variables and undermines internal validity. Moreover, the inability to perform drug-specific subgroup analyses, such as comparing pregabalin versus duloxetine within the pharmacological arms, reflects both limited data granularity and inadequate sample stratification. In the context of non-pharmacological treatments, while CBT and FIT Teens demonstrated significant benefits, there are limitations related to the nature of control conditions. For instance, in the Kashikar-Zuck trial, the comparator was a fibromyalgia education programme, which, although credible, may not have provided a robust placebo or active control equivalent to CBT in terms of therapist engagement or expectancy effects. Similarly, in the FIT Teens study, the absence of a non-treatment control group makes it difficult to disentangle the specific contributions of neuromuscular training from non-specific group effects or behavioural activation. Heterogeneity also extends to the assessment of physical activity. While the use of actigraphy in some studies provided objective data, others relied solely on self-report instruments. The discordance between subjective and objective measures further complicates interpretation, as improvements in perceived function may not correlate with measurable changes in activity levels. Additionally, few studies incorporated biomechanical or neuromuscular assessments, which are increasingly recognised as important mediators of functional outcomes in chronic musculoskeletal pain. Finally, most interventions were delivered in highly controlled research environments, with experienced clinicians and structured protocols. The generalisability of these results to community or routine care settings remains uncertain, particularly in regions with limited access to specialised paediatric pain services or multidisciplinary teams. Moreover, only articles published in English were ultimately included, although studies in other major languages were initially deemed eligible, potentially introducing a language-related selection bias. Pharmacological interventions such as NSAIDs, opioids, and injection therapies were excluded, in accordance with current international guidelines which do not recommend their use for the management of fibromyalgia [119,120]. In particular, the independent and unmonitored use of NSAIDs among adolescents could not be reliably assessed, representing a potential source of confounding. Lastly, studies with a follow-up duration of less than eight weeks were excluded, as shorter time frames are not considered sufficient to capture clinically meaningful improvements in patient-reported outcome measures, according to standards commonly adopted in the literature on juvenile fibromyalgia. Specifically, the CDI was used to assess psychological well-being, the FDI was employed to evaluate both physical and psychosocial functioning, and the VAS was used to measure pain intensity. These PROMs were selected as they represent the most consistently reported and validated tools in the context of juvenile fibromyalgia, addressing key domains such as emotional health, functional impairment, and pain perception. However, the exclusive use of these PROMs may have limited the ability to capture other relevant aspects of the patient experience, such as health-related quality of life or patient satisfaction, which are addressed by additional instruments like the PedsQL or PGIC but were inconsistently reported across the available literature.

### 4.8. Future Directions

Future research on the management of JFM should prioritise large-scale, multicentre trials with harmonised diagnostic criteria and standardised outcome measures. Longer follow-up periods are crucial for evaluating the durability of treatment effects, particularly in relation to functional restoration and engagement in physical activity. There is a clear need for head-to-head comparisons of pharmacological agents and combined approaches such as FIT Teens, as well as mechanistic studies exploring the role of central sensitisation, biomechanics, and fear of movement. Moreover, the potential use of novel agents such as low-dose naltrexone for the treatment of juvenile fibromyalgia should be investigated. Naltrexone is an opioid receptor antagonist, which, if administered in low doses, produces a decrease in pain level in patients with fibromyalgia [27]. However, data on the effectiveness, safety and optimal dosing recommendation in paediatric patients are lacking. Finally, implementation research is warranted to evaluate the feasibility, accessibility, and cost-effectiveness of integrated interventions in real-world clinical settings.

## 5. Conclusions

JFM is a definite clinical challenge, both in terms of diagnosis and management. Pharmacological treatments such as duloxetine, pregabalin, and milnacipran have been studied in adolescents, but their overall benefit remains modest and often limited to pain reduction, with inconsistent effects on function and mood. On the other hand, non-pharmacological interventions, particularly cognitive-behavioural therapy, appear more effective in improving functional outcomes and emotional well-being, although their impact on pain is less clear. Emerging integrative models, such as the FIT Teens programme, offer a promising direction by combining psychological support with targeted physical training. Despite these developments, the available evidence remains scarce, and high-quality, long-term studies are urgently needed. Future research should aim to clarify the most effective combinations of treatment components and define individualised pathways for this underserved patient population.

## Figures and Tables

**Table 1:** Strings used for the search in each database.

Database	Search String
PubMed	(“juvenile fibromyalgia”\((MeSH Terms)) OR “juvenile fibromyalgia” OR “adolescent fibromyalgia”) AND (“drug therapy”\((MeSH Terms)) OR pharmacologial OR antidepressants OR pregabalin OR duloxetine OR milnacipran) AND (“non-pharmacological” OR “cognitive behavioral therapy” OR exercise OR physiotherapy OR “multidisciplinary approach”) AND (randomized controlled trial\((pt)) OR “RCT” OR “randomised controlled trial”)
Web of Science	TS = (“juvenile fibromyalgia” OR “adolescent fibromyalgia”) AND TS = (“pharmacological” OR “drug therapy” OR duloxetine OR pregabalin OR milnacipran) AND TS = (“non-pharmacological” OR “cognitive behavioral therapy” OR exercise OR physiotherapy OR “multidisciplinary approach”) AND TS = (“randomized controlled trial” OR “RCT”)
Embase	(‘juvenile fibromyalgia’/exp OR ‘juvenile fibromyalgia’ OR ‘adolescent fibromyalgia’) AND (‘drug therapy’/exp OR pharmacological OR duloxetine OR pregabalin OR milnacipran) AND (‘non drug therapy’/exp OR ‘cognitive behavioral therapy’ OR exercise OR physiotherapy OR ‘multidisciplinary care’) AND (‘randomized controlled trial’/exp OR ‘randomised controlled trial’ OR RCT)

**Table 2:** Generalities of the included studies (FIT: Fibromyalgia Integrative Training; CBT: cognitive-behavioural therapy).

Author, Year	Journal	Follow-Up (*Weeks*)	Treatment	Mean Age	Female (%)	Patients (*n*)
Arnold et al., 2015 [29]	*Pediatr Rheumatol Online J*	8	Milnacipran (50 to 100 mg/daily)	15.7	90	96
			Milnacipran (50 to 100 mg/daily)/Placebo	15.0	60	20
Arnold et al., 2016 [30]	*Pediatr Rheumatol Online J*	15	Pregabalin (75 to 450 mg/daily)	14.6	86	54
			Placebo	14.7		53
Black et al., 2021 [31]	*Clin J Pain*	8	CBT	15.3	90	20
			FIT Teens			20
Hengartner et al., 2021 [32]	*Int J Risk Saf Med*	13	Duloxetine (30 to 60 mg/daily)	15.7	80	91
			Placebo	15.3	70	93
Kashikar-Zuck et al., 2012 [33]	*Arthritis Rheum*	24	CBT	15.2	95	57
			Education treatment	14.9	90	57
Kashikar-Zuck et al., 2013 [34]	*Arthritis Care Res (Hoboken)*	9	CBT	15.2	94	33
			Education treatment			35
Kashikar-Zuck et al., 2018 [35]	*J Pain*	12	CBT		90	19
			FIT Teens			17
Upadhyaya et al., 2019 [36]	*Pediatr Rheumatol Online J*	13	Duloxetine (30 to 60 mg/daily)	15.7	80	91
			Placebo	15.3	70	93

**Table 3:** Overview of the main results (FIT: Fibromyalgia Integrative Training; CBT: cognitive-behavioural therapy; PGIS: Patient Global Impression of Severity, Pediatric Quality of Life Inventory; PedsQL: Generic Core Scales, Multidimensional Fatigue Scale, MASC: Multidimensional Anxiety Scale for Children; CDI: Children’s Depression Inventory, AEs: adverse events; LTR: loss of therapeutic response; NRS: numeric rating system; PGIC: patient global impression of change; parent GIC: parent global impression of change (parent GIC), FIQ-C: Fibromyalgia Impact Questionnaire for children; FDI: Functional Disability Inventory; VAS: visual analog scale; HRQOL: health-related quality of life; TSK-11: The Tampa Scale for Kinesiophobia; PCS-C; 40: Pain Catastrophizing Scale for Children; PPQ: Pediatric Pain Questionnaire; CGI-severity: Clinical Global Impression of severity scale; CGI-severity: mental illness: Clinical Global Impression of severity for mental illness scale; FDI-child: Functional Disability Inventory-child version scale; FDI-parent: Functional Disability Inventory-parent version scale; BPI: Brief Pain Inventory).

Author, Year	Treatment	Outcome of Interest	Main Results
Arnold et al., 2015 [29]	Milnacipran (50 to 100 mg/daily)	Pain, PGIS, PedsQL: Generic Core Scales, Multidimensional Fatigue Scale, MASC, CDI, AEs, vital signs (blood pressure, Heart rate) body weight, electrocardiograms, and laboratory tests, LTR	Mean improvements in pain, global disease severity, quality of life, and fatigue symptoms at the end of both open-label periods. Nausea, headache, vomiting, and dizziness as most common reported adverse events. Mean increases in heart rate and blood pressure.
Arnold et al., 2016 [30]	Pregabalin (75 to 450 mg/daily) vs. placebo	primary efficacy outcome: change in mean pain score based on the subject’s daily pain diaries (NRS). Secondary efficacy outcomes: mean pain score at each week, from daily pain diaries with a 24-h recall period; the change in mean pain score at week 15 with a 1-week re- call period; PGIC, change in sleep quality score at endpoint and at each week, parent GIC, FIQ-C.	Not significant improvement in mean pain score at endpoint. Significant improvements with pregabalin versus placebo in secondary outcomes of change in pain score by week and patient global impression of change. No significant improvement in other secondary outcomes measuring pain, sleep, and FM impact. Safety in line with pregabalin’ known profile in adults
Black et al., 2021 [31]	CBT vs. FIT Teens	Isokinetic hip and knee strength, dynamic postural stability, and 3-D motional analysis of functional tasks conducted on a standardized drop vertical jump task.	Improvements in hip abduction strength and greater external hip rotation in the FIT Teens group. Decreased hip adduction in the FIT Teens group.
Hengartner et al., 2021 [32]	Duloxetine (30 to 60 mg/daily) vs. placebo	Severe treatment-emergent psychiatric adverse event including all AE related to suicidality and other psychiatric disorders, treatment discontinuation due psychiatric adverse event	Significant treatment-emergent suicidal ideation and behaviour with duloxetine. The incidence of severe treatment-emergent psychiatric adverse events significantly higher in duloxetine group.
Kashikar-Zuck et al., 2012 [33]	CBT	Primary outcome: FDI. Secondary outcomes: CDI, VAS, tender point sensitivity, physician’s global assessment on a 0–10-cm VAS, HRQOL using the PedsQL Generic Core Scales and PedsQL Rheumatology Module, sleep quality. AEs.	CBT was superior to FM education in reducing the primary outcome of functional disability. Depression symptoms reduction was significant for both groups. Reduction in pain was not significant in either group
Kashikar-Zuck et al., 2013 [34]	CBT vs. Education	Actigraphy, FDI, CDI	Self-reported functioning improved in the CBT group but no significant changes were seen in either group for activity counts, sedentary, moderate, or vigorous activity. The CBT showed lower peak and light activity.
Kashikar-Zuck et al., 2018 [35]	CBT vs. FIT Teens	Primary outcomes: VAS, FDI. Secondary outcomes: CDI, TSK-11, PCS-C; 40, Aes	FIT Teens group had significantly greater decreases in pain. FIT Teens reported significant improvements in disability, but did not differ from CBT at the 3-month
Upadhyaya et al., 2019 [36]	Duloxetine (30 to 60 mg/daily) vs. placebo	Primary outcome: mean change in 24-h average pain severity of BPI. Secondary outcomes: BPI-modified short form: adolescent version severity and interference scores, PPQ (pain right now, worst pain, and average pain items), CGI-severity: overall, CGI-severity: mental illness, FDI-child, FDI-parent, CDI, Multidimensional Anxiety Scale for Children, treatment response (≥30%, ≥50% reductions on BPI average pain severity), AEs, laboratory values, height, weight, vital signs, and electrocardiograms.	Change in BPI average pain severity was not different between duloxetine and placebo. Duloxetine was better in treatment response (≥30% and ≥50% reductions on BPI average pain severity) and improvement of the general activity and relationships items on the BPI interference subscale. Onset of treatment-emergent adverse event more frequent in the duloxetine group

## Data Availability

The original contributions presented in this study are included in the article. Further inquiries can be directed to the corresponding author.
